# Variations in Cortical Oxygenation by Near-Infrared Spectroscopy According to Head Position after Acute Stroke: The Preliminary Findings of an Observational Study

**DOI:** 10.3390/jcm13133914

**Published:** 2024-07-03

**Authors:** Ilaria Casetta, Anna Crepaldi, Michele Laudisi, Andrea Baroni, Jessica Gemignani, Sofia Straudi, Fabio Manfredini, Nicola Lamberti

**Affiliations:** 1 IRCCS San Camillo Hospital, 30126 Venice, Italy; cti@unife.it; 2 Unit of Nephrology, University Hospital of Ferrara, 44124 Ferrara, Italy; anna.crepaldi@edu.unife.it; 3 Unit of Neurology, University Hospital of Ferrara, 44124 Ferrara, Italy; michele.laudisi@unife.it; 4 Unit of Rehabilitation Medicine, University Hospital of Ferrara, 44124 Ferrara, Italy; andrea.baroni@unife.it (A.B.); sofia.straudi@unife.it (S.S.); 5 Department of Neuroscience and Rehabilitation, University of Ferrara, 44124 Ferrara, Italy; nicola.lamberti@unife.it; 6 Department of Developmental Psychology and Socialization, University of Padova, 35131 Padova, Italy; jessica.gemignani@unipd.it; 7 Program of Vascular Rehabilitation and Exercise Medicine, University Hospital of Ferrara, 44124 Ferrara, Italy

**Keywords:** stroke, rehabilitation, head position, fNIRS

## Abstract

**Background**: After ischemic stroke, there is no general consensus on the optimal position for the head of patients in the acute phase. This observational study aimed to measure the variations in cortical oxygenation using noninvasive functional near-infrared spectroscopy (fNIRS) at different degrees of head positioning on a bed. **Methods**: Consecutive ischemic stroke patients aged 18 years or older with anterior circulation ischemic stroke within 48 h of symptom onset who could safely assume different positions on a bed were included. A 48-channel fNIRS system was placed in the bilateral sensorimotor cortex. Then, the bed of each patient was moved into four consecutive positions: (1) seated (90° angle between the head and bed surface); (2) lying at 30°; (3) seated again (90°); and (4) lying flat (0°). Each position was maintained for 90 s; the test was conducted 48 h after stroke onset and after 5 ± 1 days. The variations in oxygenated hemoglobin in the global brain surface and for each hemisphere were recorded and compared. **Results**: Twenty-one patients were included (males, n = 11; age, 79 ± 9 years; ASPECTS, 8 ± 2). When evaluating the affected side, the median oxygenation was significantly greater in the lying-flat (0°) and 30° positions than in the 90° position (*p* < 0.001 for both comparisons). No significant differences between the supine position and the 30° position were found, although oxygenation was slightly lower in the 30° position than in the supine position (*p* = 0.063). No differences were observed when comparing recanalized and nonrecanalized patients separately or according to stroke severity. The evaluation conducted 5 days after the stroke confirmed the previous data, with a significant difference in oxygenation at 0° and 30° compared to 90°. **Conclusions**: This preliminary study suggested that there are no substantial differences in brain oxygenation between the lying-flat head position and the 30° laying position.

## 1. Introduction

Stroke, or a cerebrovascular accident in the brain lasting more than 24 h, is the second leading cause of death and the third leading cause of death and disability combined worldwide [1]. Stroke significantly impacts on both the physical and mental health of affected patients and their quality of life, which is directly related to the severity of the episode and the number of comorbidities that the patient suffers [2].

Several studies have attempted to identify the best head position after acute ischemic stroke (AIS). Therapeutic head positioning plays a part in disease management, as it may have beneficial effects on the brain physiology of these patients [3]. For critically ill patients, a semirecumbent position with head elevation at an angle of 30° is advised, allowing for enteral nutrition and reducing the risk of pneumonia [4]. Moreover, several studies have demonstrated that the elevation of the head of the bed lowers intracranial pressure, but, at the same time, reflects concomitant changes in systemic blood pressure, keeping cerebral perfusion unchanged [5,6]. On the other hand, brain oxygenation should be considered a target that can affect the outcome of patients with brain injury [3,7]. Indeed, brain oxygenation can be reduced when patients are in an upright position, considering its effect on pressure [3], but studies on traumatic brain injury patients demonstrated that the elevation of the head from the supine to 30° position reduced intracranial pressure without changes in brain oxygenation [8,9,10]. Nevertheless, flat positioning improves the mean blood flow velocity (MFV) in AIS patients, as demonstrated by transcranial Doppler (TCD) studies [11,12,13,14,15,16,17]. The clinical significance of these findings has not been elucidated. The international multicenter Head Position in Acute Stroke Trial (HeadPoST) revealed no differences in disability outcomes after acute stroke between patients assigned to a lying-flat position and patients assigned to a sitting-up position with the head elevated to at least 30 degrees for 24 h in the total population of AIS patients and in patients with moderate–severe AIS [18,19]. Currently, evidence to recommend a specific head position in the acute phase of AIS is scarce, leading to a lack of general consensus on the best position for these patients in clinical practise [20,21]. Hence, further studies are needed to determine the optimal head position in the acute phase of stroke. Transcranial Doppler, the most commonly used method for studying cerebral flow in acute stroke patients at the bedside, is a user-dependent technology, and the absence of a temporal window for TCD examination occurs in 5 to 15% of patients, preventing the possibility of measuring the MFV in this subgroup of patients [22].

Nevertheless, other neuroimaging methods for analyzing blood flow are available. Functional magnetic resonance imaging (fMRI) is the gold standard for the in vivo imaging of the human brain [23], and functional near-infrared spectroscopy (fNIRS) has gained increasing popularity for studying brain function. Compared to functional magnetic resonance imaging (fMRI), fNIRS has several advantages, including measurements of concentration changes in both oxygenated and deoxygenated hemoglobin, finer temporal resolution, and an ease in administration, as well as disadvantages, the most prominent of which include inferior spatial resolution and decreased signal-to-noise ratio [23]. However, to the best of our knowledge, no previous techniques have been employed to study head positioning after acute stroke.

This pilot study aimed to explore the potential of fNIRS for assessing cerebral oxygenation in AIS patients according to different head positions.

## 2. Materials and Methods

This observational study was conducted at the Unit of Clinical Neurology at the University Hospital of Ferrara (Ferrara, Italy). This study was approved by the local ethics committee (445/2018/Sper/AOUFe; approved on 18 July 2018). Written informed consent was obtained from the patients or their approved representatives. This manuscript is reported according to the STROBE guidelines.

### 2.1. Inclusion Criteria

We assessed for possible enrollment all consecutive ischemic stroke patients admitted to the Stroke Unit of the University Hospital of Ferrara. Patients were eligible for inclusion if they were 18 years of age or older, presented with anterior circulation ischemic stroke within 48 h of symptom onset, and could be safely positioned lying flat (0°) and upright at 30° and 90°. The exclusion criteria were dysphagia or any contraindication to assume the abovementioned positions.

### 2.2. Experimental Task

The patients were equipped for the entire duration of the experimental task with a continuous wave NIRS system (NIRScout, NIRx, Glean Head, NY, USA) with a temporal resolution of 3.47 Hz. The NIRS optical probe consists of 16 laser sources at two different wavelengths (690 and 830 nm, emitting ~10 mW each) and 16 detectors placed on the scalp to cover most of the frontal and parietal lobes. The NIRScap layout is set on a 10-5 position recommended by the NIRx user manual for NIRScaps and probes with a topographic setting: this particular configuration is the standard layout for motor measurement with NIRS that includes short channels to record hemodynamic changes in the middle frontal, superior frontal, precentral, and postcentral gyri.

This configuration allows for 48 different source-detector pairs (channels) with a distance of 3 cm between sources and detectors, with a near infrared light penetration depth of approximately 1.52 cm. The configuration included 7 short channels, where the source–detector distance was 7 mm and the NIR light penetration depth was 4–5 mm. The use of short channels is implemented to exclude possible confounding factors, such as scalp vascularization from detection [24]. To position the channels correctly on the head and enable correct detection, a specific headset with a semielastic mesh structure suitable for being placed comfortably on the head is needed. A Velcro fastener under the patient’s chin ensured the stability of the headset, so that it could be easily adapted to each subject. To correctly position the cuff, the “reference point” (RP), located at the top of the cuff itself, was considered the reference. The RP is a conventional point located at the intersection of the distance between inion and the nasion and the interear distance. These two distances were measured with a tape measure, and the RP on the patient’s skin was marked with a felt-tip pen to position the cuff precisely. This procedure is essential because it allows the same portions of the brain to be assessed in different evaluations, and at the same time standardizes the assessment between different patients.

Once put on, another black fabric headset covered the first one for the correct reception of the channels, which could be disturbed by the presence of external light sources. Once positioned, both headsets remained stationary throughout the duration of the evaluation. A monitor connected to the fNIRS acquisition server was also placed on the table for the correct timing of the task and the correct insertion of the markers at every posture’s modification.

The data collection protocol was performed as follows:

- Part 1. Recording starts when the headset is correctly positioned, and the detection system is correctly calibrated automatically using NIRStar acquisition software version 15.6.
- Part 2. The first marker is placed upon reaching the upright position on the bed by forming an approximately 90° angle between the trunk and the outstretched lower limbs. The position is held for 90 s before proceeding to the next position. Pillows are placed on the back to support the posture, and assistance is provided to maintain the position with as little muscle effort as possible.
- Part 3: The second marker is placed when the subject is positioned to form a 30° angle between the trunk and the horizontal plane of the bed, maintaining the posture for an additional 90 s.
- Part 4: The third marker is placed when the patient is positioned again sitting up, forming a 90° angle between the trunk and lower limbs for an additional 90 s.
- Part 5: The fourth and last marker is placed when the patient is in the supine position (0° angle). The recording ended after the last 90 s were recorded.

**Figure 1:**
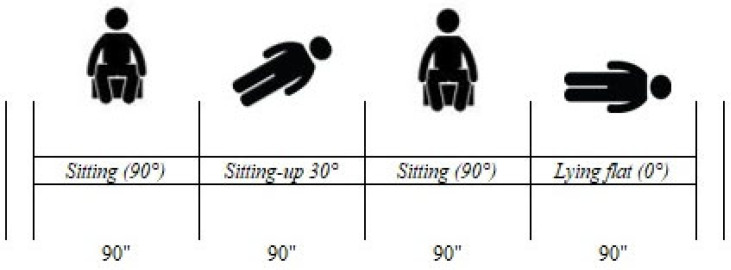
Graphical representation of the experimental task.

The procedure took a total of 7 min and 30 s, and the entire protocol, including the placement of the NIRS cuff, the calibration of the machine, and the removal of the cuff at the end of the recording, took approximately 12 to 15 min. The protocol was performed twice: within 48 h after stroke and between 5 and 6 days after the ischaemic event. The same skilled operator recorded all detections for the entire duration of the study. A graphical representation of the intervention is shown in Figure 1.

### 2.3. Data Analysis

The measured data were converted using MATLAB R2021a: the detection of the hemodynamic state was possible due to the use of the Homer2 application. Homer2 allows for the extraction and correct preprocessing of the data by applying filters that allow for the removal of motion artefacts, i.e., all elements that interfere with the signal. These can be due to the movements of the analyzed subject as well as normal physiological parameters, such as heart rate, blood pressure, respiratory rate, and blood flow to the scalp (regression using short channels).

The filters were chosen according to their properties, study design, and guidelines reported by the scientific community.

The filters used in preprocessing are described below:

- hmrIntensity2OD: This converts light intensities into optical densities.
- hmrSSR: This allows for short-separation regression. By using short channels, it is possible to separate the superficial extracerebral vascular component, i.e., of the scalp, from the actual cortical vascular component [25].
- hmrOD2Conc: This converts optical densities into concentrations. The input data are the obtained optical densities, the SD (source–detectors) structure and the partial pathlength factor (ppf) for each wavelength. The typical value of ppf is ~6 for each wavelength if the absorption variation is uniform over the measured volume of tissue.
- hmrMotionCorrectPCA: This function uses the PCA filter to filter out only those segments identified as motion artefacts. The input data include nSV, which is the number of principal components to be removed from the data (0.80). If this number is less than 1, the filter removes the first n components of the data, removing a fraction of the variance up to nSV. Several authors have used this procedure to remove extracerebral components from signals, thus removing systemic interference [26].
- hmrBandpassFilt: A bandpass filter is applied. This procedure involves the exclusion of frequencies not between two chosen values, namely, a high-pass filter (0.01) and a low-pass filter (0.5). The frequency range is set to preserve the frequency of the stimulus in relation to the task [27].

The files were finally converted to the “.csv” format using MATLAB scripts.

The data were then imported into an Excel workbook. The oxygenated hemoglobin (O_2_Hb) values were analyzed, as these values were the most significant for the investigation. For each file, all channels were averaged for each frame, and the recorded markers are reported. Values high to the tenth and above are excluded from the average, as they are not clinically significant and are considered confounding factors of the true trend.

For analysis, the changes from baseline of the mean values calculated 90 s after the marker were then isolated, and a 2D line graph was projected to verify the trend at the four positions. The calculated parameter for each position was the area under the curve, or the sum of all O_2_Hb values collected for the duration of the task.

### 2.4. Outcomes

The demographic, clinical, and radiological characteristics of the patients, including sex, age, preadmission disability (modified Rankin scale, mRS), vascular risk factors, medication, etiology of stroke categorized according to the TOAST classification, severity of stroke at admission (National Institutes of Health Stroke Scale—NIHSS), Alberta Stroke Program Early CT score (ASPECTS), site of vessel occlusion, and type of treatment (intravenous thrombolysis (IVT), endovascular procedure with or without IVT, and best medical therapy), were collected by the attending neurologist. Data about recanalization after treatment were also recorded.

The primary outcome of the study was the feasibility of assessing changes in cerebral perfusion in relation to body position following ischemic stroke using fNIRS. The secondary outcome concerns the assessment of hemodynamic changes in terms of O_2_Hbin different postures using short-channel analysis and the variations that could occur in the two different timepoints of collection after AIS.

### 2.5. Statistical Analysis

The baseline characteristics of the patients are reported as the mean ± standard deviation or median (interquartile range) according to the data distribution. For data analysis, the area under the curve per position trend was calculated. The values found were summed for each marker, and the mean and standard deviation were calculated for each position for descriptive statistics. The O_2_Hb trends in the different postures are presented, including the short channel analysis. Within-patient variations were assessed using a paired samples t-test or Wilcoxon signed-rank test, as appropriate. The consistency of the two measurements over time was verified with a Passing–Bablok regression. A *p* value < 0.05 was considered to indicate statistical significance. Data analyses were performed with MedCalc^®^ Statistical Software version 22.021 (MedCalc Software Ltd., Ostend, Belgium).

## 3. Results

A total of 21 patients (11 males, mean age 79 ± 9 years) were included in this study.

The median NIHSS score at baseline was 16 (IQR 9–20.5). Twelve patients had M1 occlusion (six right and six left), six patients had left M2 occlusion, and two patients had left M3 occlusion. Finally, one patient had a left internal carotid terminus occlusion. The median ASPECTS was 8 (IQR 7–10). The clinical and demographic characteristics of the included patients are summarized in Table 1.

Six patients were not eligible for any revascularization treatment, seven were treated with IVT, and the remaining underwent endovascular thrombectomy with or without previous IVT. Complete or near-complete recanalization was proven in 14 (67%) patients.

### 3.1. fNIRS Data Collection

fNIRS data were collected by the same skilled operator throughout the entire study duration. The procedure lasted approximately 15 min and included explanation, instrument set up, and data collection. No adverse events were reported during the data collection, and no patient complained of pain or discomfort during the procedure.

The data analysis was performed for all the traces collected following the steps reported in the Section 2.

**Figure 2:**
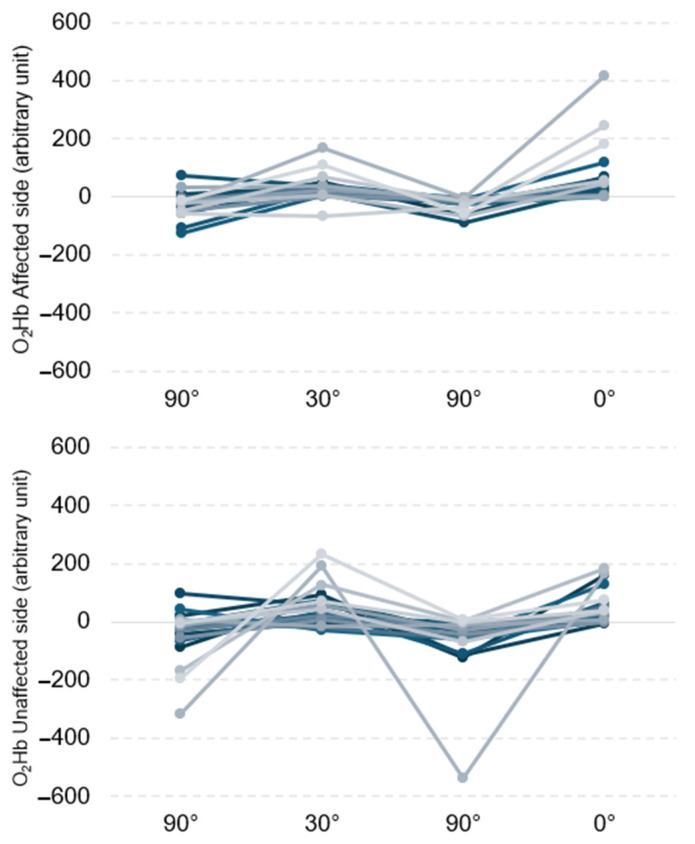
Individual trends (each line represents one patient) of O_2_Hb in both affected (top) and unaffected (bottom) hemispheres.

During the first execution of the experimental task, considering the entire brain area and both hemispheres, significantly higher values of O_2_Hbwere observed for the 0° and 30° positions than for the 90° position. The data are reported in Table 2 and are graphically represented in Figure 2.

A superimposable trend was observed when the experimental task was repeated 5 ± 1 days after the stroke, confirming significantly greater O_2_Hb values for the 30° and 0° positions than for both the 90° positions. The data are reported in Table 3.

### 3.2. fNIRS Measurement Agreement

**Figure 3:**
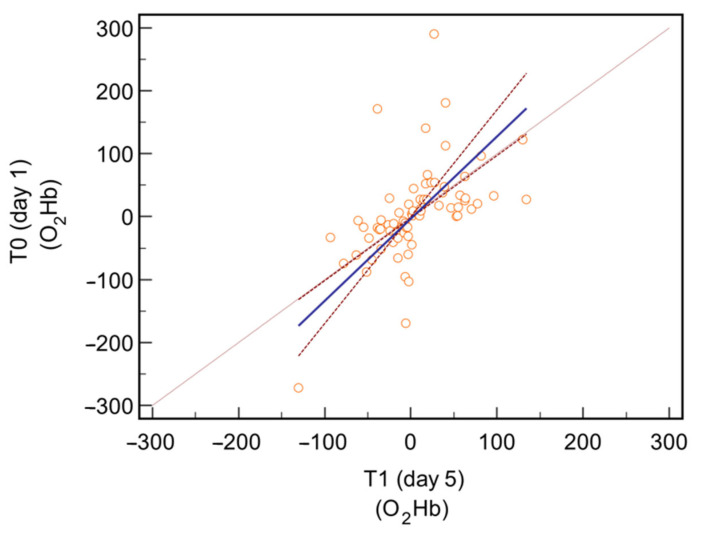
Passing–Bablok regression comparing the O_2_Hb values for the entire brain area collected at T0 and at T1.

When comparing the measurements of O_2_Hb on both sides, a mean difference of 0.3 units (confidence interval −13.29 to 13.92; *p* = 0.96) was observed between the values collected at T0 and those collected at T1. The following Passing–Bablok regression confirmed no significant deviations from linearity (*p* = 0.90, residual standard deviation 56.37 units). Figure 3.

### 3.3. Within-Subject Variations over Time

**Figure 4:**
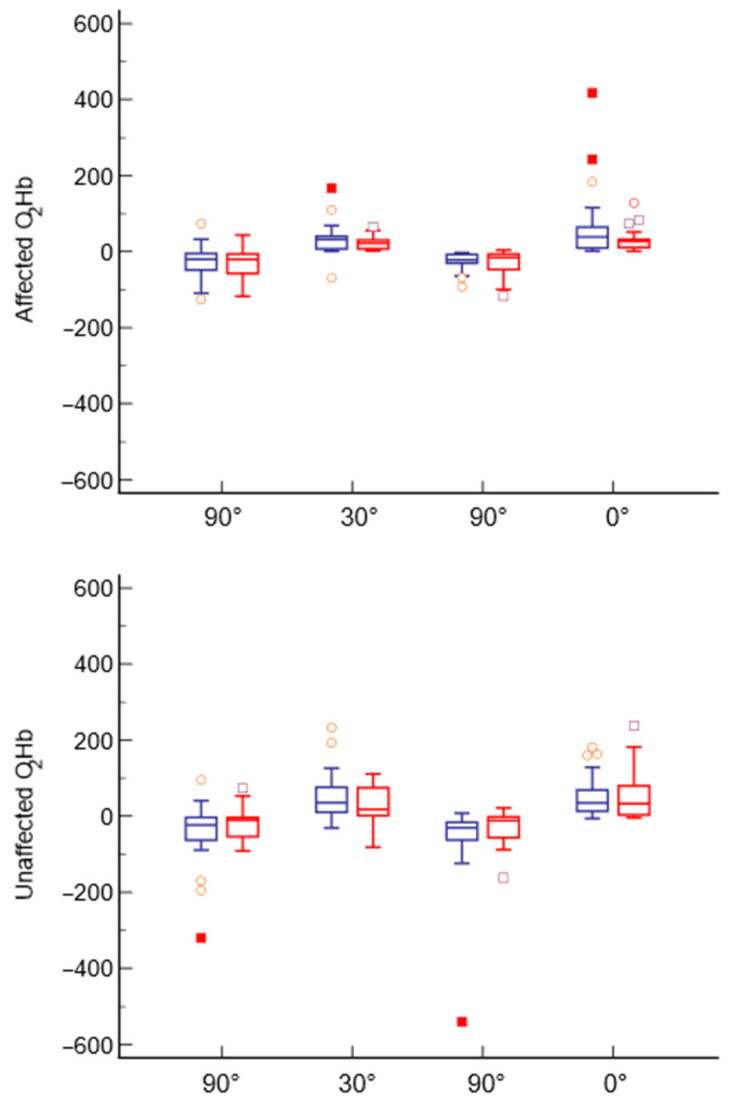
Comparison of the four different positions collected at both sides at T0 (blue) and T1 (red).

When comparing the values of both hemispheres from T0 to T1, no significant differences were observed for any of the positions scheduled in the experimental task. The comparison is reported in Figure 4.

### 3.4. Comparison between Recanalized and Nonrecanalized Patients

**Figure 5:**
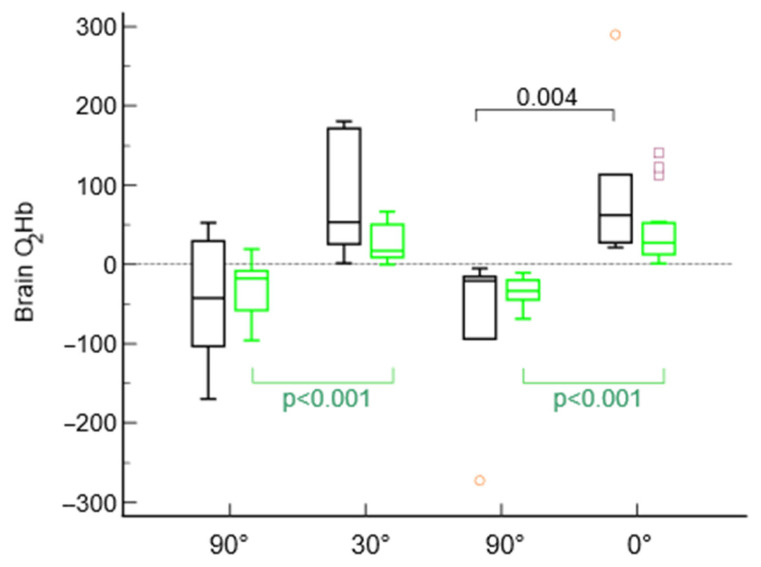
Comparison of the four different positions collected in the entire brain area in recanalized patients (green) and nonrecanalized patients (black).

When analyzing recanalized and nonrecanalized patients separately, we found that in recanalized subjects, O_2_Hb was significantly greater in the lying-flat position and in the 30° sitting up position than in the 90° position (*p* < 0.001 for both comparisons), while there were no significant differences between the 30° and 0° recumbency positions (*p* = 0.22). Conversely, in nonrecanalized patients, although the O_2_Hb was greater at 30° and 0°, there were no significant differences between the analyzed body positions (Figure 5).

### 3.5. Comparison According to Stroke Severity

When evaluating patients stratified according to baseline stroke severity, we found no significant differences in the O_2_Hb levels between the different body positions in mild stroke patients, while in patients with moderate to severe stroke, we confirmed that the O_2_Hb was significantly greater at 0° and 30° than in the sitting position (*p* < 0.001 for both comparisons). Compared with the 30° position, the flat position was significantly associated with the highest O_2_Hb (41.1 ± 37.4 vs. 32.4 ± 31.6; *p* = 0.023).

## 4. Discussion

This pilot study revealed that the collection of brain oxygenation according to the head position with the patient lying in bed is both feasible and does not cause any discomfort for the subject. From a hemodynamic point of view, the data collected showed no substantial differences in brain oxygen saturation between the lying-flat head position and the 30° position, even if patients with more severe stroke showed an improvement in O_2_Hb levels from a height of 30° to 0° recumbency.

Cerebral blood flow is critically important for maintaining the perfusion of the ischemic penumbra in patients with AIS. A sitting-up position can reduce intracranial pressure and the risk of aspiration pneumonia, but it may affect cerebral perfusion by decreasing cerebral blood flow (CBF) in salvageable tissue, especially when cerebral autoregulation is impaired [28]. Conversely, a flat position allows for enhanced blood flow in the collateral and leptomeningeal circulation [12,14,29].

Small observational studies using transcranial Doppler ultrasonography (TCD) in patients with anterior circulation stroke suggested that an increase in cerebral mean flow velocity (MFV) in the flat position, due to gravitational force and/or better recruitment of collateral vessels, could improve residual CBF [12,14,17].

A systematic review and meta-analysis of observational studies evaluating differences in cerebral MFV between the lying-flat and sitting positions in anterior circulation stroke patients concluded that the MFV increased significantly on the side affected by stroke when the patients were positioned in a flat-head position at 0 or 15° compared to an upright-head position at 30° [17]. However, there remains uncertainty (7) over the relevance of these observations to clinical outcomes [17,18] and some important adverse events, such as intracranial hypertension and aspiration pneumonia [30,31,32]. Regarding this latter aspect, a low incidence of pneumonia attributable to position was observed in stroke patients lying flat after intravenous thrombolysis, suggesting that avoiding this position due to concerns about subsequent pneumonia may not be justified [33].

Although a 30° head elevation has been suggested for patients with neurological conditions such as severe head trauma [30,31], this position could be more advantageous for patients with increased intracranial hypertension. However, considering that stroke is a heterogeneous condition, it is unlikely that one single optimal position will be optimal for all patients. Most likely, different positions are preferred according to stroke severity and recanalization status. Positioning should then be defined on an individual basis and eventually modified for the same patients according to their changing clinical conditions [13] or in relation to provocative actions or specific protocols (e.g., moderate intermittent hypoxia conditioning) [34].

In this context, an ideal method for routine clinical use should be noninvasive, easy to accomplish, and easily repeatable. Both TCD and fNIRS noninvasively measure regional cerebral oxygenation (rSO2) and cerebral blood flow (CBF) velocity (CBFV), respectively, as proxies of cerebral perfusion.

Evaluating the CBFV of the MCA may not be a reliable index for assessing cerebral perfusion, particularly at the microcirculatory level, which plays a pivotal role in the extension of the ischemic penumbra. Moreover, TCD is highly operator-dependent, and in approximately 20% of patients, no appropriate acoustic window can be found, and the CBF cannot be estimated on the affected side when the vessel is permanently occluded [13]. Near-infrared spectroscopy, which also allows for the noninvasive measurement of rSO2, has gained increasing interest in the field in recent years [23].

A study employing both TCD and fNIRS methodologies, carried out on patients with mild to moderate stroke, showed that upright positioning does not significantly reduce CBF or frontal cerebral oxygenation surrogates during the subacute phase of stroke [15].

Conversely, Truijen et al. applied both methods (TCD and fNIRS) to assess cerebral perfusion in relation to head posture in patients with anterior circulation stroke and provided evidence that cerebral autoregulatory performance in these patients affects the cerebrovascular response to changes in head position, favouring head-of-bed lowering in subjects with impaired autoregulation [35].

This study has several limitations, including its limited sample size. First, the limitations of the fNIRS technique, especially those related to the semiquantitative nature of the data and the depth of light penetration, need to be acknowledged. Second, the analysis time was quite long, and expert personnel were required to analyze fNIRS data. Finally, the inclusion of patients with anterior circulation ischaemic stroke may only reduce the external validity of the results, considering that cerebral infarcts in the territory of the anterior cerebral artery have been reported with a better prognosis than infarcts in the territory of the middle cerebral artery [36] [vb PMID: 19589132].

## 5. Conclusions

Overall, the available data indicate that the fNIRS technique allows for a feasible assessment of cortical oxygenated hemoglobin variations in different postures in patients with AIS, and the effects of changes in head positioning on cortical perfusion can be monitored and quantified. Our preliminary data suggested that there are no substantial differences in brain oxygenation between the lying-flat head position and the 30° laying position. This enables, without a generic optimal recommendation in patients affected by AIS [21], individualized management based on bedside monitoring to optimize perfusion. In this context, fNIRS, despite its limited detection depth, can be regarded as a valuable option because it is a real-time, noninvasive, easily repeatable, investigator-independent, and easily portable tool for monitoring brain perfusion [37,38]. Further studies are warranted to confirm these preliminary data, particularly focusing on the estimation of real-time brain oxygenation according to head position for longer periods and on the identification of oxygenation values in the brain areas affected by stroke.

## Figures and Tables

**Table 1:** Characteristics of the population included in this study.

	Population (n = 21)
Women (N, %)	10 (48%)
Mean + SD age	79 ± 9
Hypertension (N, %)	18 (86%)
Diabetes (N,%)	3 (14%)
Atrial fibrillation (N, %)	12 (57%)
Dyslipidemia (N, %)	9 (43%)
Ischemic heart disease (N, %)	8 (38%)
Smoking (N, %)	6 (29%)
Baseline NIHSS (median, IQR)	16 (9–21)
Baseline ASPECTS (median, IQR)	8 (7–10)

**Table 2:** O_2_Hb values for the different brain regions according to head position.

	T0, 90° (First)	T0, 30°	T0, 90° (Second)	T0, 0°	*p* Value
Whole brain area	−35.70 ± 51.23	42.77 ± 49.49 †*	−45.71 ± 56.53	59.28 ± 68.22 †*	<0.001
Affected hemisphere	−25.48 ± 43.03	33.39 ± 45.19 †*	−26.25 ± 24.50	68.56 ± 100.71 †*	<0.001
Unaffected hemisphere	−45.92 ± 89.46	52.15 ± 66.45 †*	−65.16 ± 115.06	53.05 ± 56.97 †*	<0.001

† *p* < 0.05 with respect to T0, 90° (first); * *p* < 0.05 with respect to T0, 90° (second).

**Table 3:** Values of O_2_Hb for the different brain regions according to head position collected at 5 ± 1 days.

	T0, 90° (First)	T0, 30°	T0, 90° (Second)	T0, 0°	*p* Value
Whole brain area	−25.63 ± 24.64	28.87 ± 27.85 †*	−28.98 ± 34.29	46.17 ± 41.00 †*	<0.001
Affected hemisphere	−32.51 ± 39.18	23.32 ± 18.85 †*	−30.02 ± 34.36	33.42 ± 31.79 †*	<0.001
Unaffected hemisphere	−18.76 ± 44.54	34.42 ± 48.00 †*	−27.94 ± 45.23	58.92 ± 69.94 †*	<0.001

† *p* < 0.05 with respect to T0, 90° (first); * *p* < 0.05 with respect to T0, 90° (second).

## Data Availability

The dataset used in this study is available upon request to the corresponding author.
